# UPLC-MS/MS Technology for the Quantitative Methodology and Pharmacokinetic Analysis of Voxtalisib in Rat Plasma

**DOI:** 10.3389/fphar.2022.914733

**Published:** 2022-06-14

**Authors:** Qingqing Li, Ya-nan Liu, Jing Wang, Yingying Hu, Jinyu Hu, Ren-ai Xu, Liu Shao, Lianguo Chen

**Affiliations:** ^1^ The First Affiliated Hospital of Wenzhou Medical University, Wenzhou, China; ^2^ Institute of Molecular Toxicology and Pharmacology, School of Pharmaceutical Sciences, Wenzhou Medical University, Wenzhou, China; ^3^ Chongqing University Cancer Hospital, Chongqing, China

**Keywords:** voxtalisib, quantitative methodology, pharmacokinetic analysis, UPLC-MS/MS technology, rat plasma

## Abstract

Voxtalisib, is a specific, effective, and reversible dual inhibitor, which inhibits both pan-class I phosphoinositide 3-kinase (PI3K) and mechanistic target of rapamycin (mTOR). To date, voxtalisib has been studied in trials for melanoma, lymphoma, glioblastoma, breast cancer, and other cancers. In this study, a highly sensitive and rapid ultra-performance liquid chromatography tandem mass spectrometry (UPLC-MS/MS) technology was applied to the quantitative methodology and pharmacokinetic analysis of voxtalisib in rat plasma. After protein precipitation of the analyte by acetonitrile, the chromatographic separation was performed by gradient elution on an Acquity BEH C18 column (2.1 mm × 50 mm, 1.7 μm) with acetonitrile (solvent A) and 0.1% formic acid (solvent B) as the mobile phase. In the positive ion mode, the mass transfer detection of the analyte and IS was *m/z* 270.91 > 242.98 and *m/z* 572.30 > 246.10, respectively. In the concentration range of 1–2000 ng/ml, a good linear relationship of voxtalisib was successfully established by the UPLC-MS/MS technology, and the lower limit of quantification (LLOQ) of the analyte was identified as 1 ng/ml. Intra-day and inter-day precisions for voxtalisib were 7.5–18.7% and 13.0–16.6%, respectively, and the accuracies were in the ranges of −14.0–2.0% and −7.2–3.1%, respectively. The matrix effect, extraction recovery, carryover and stability of the analyte were all in compliance with the acceptance criteria of bioassays recommended by FDA. Finally, the pharmacokinetic profile of the analyte had been availably studied by the UPLC-MS/MS bio-analytical method after rats were treated by intragastric administration with voxtalisib (5 mg/kg). The results indicated that the UPLC-MS/MS technology can effectively and quickly quantify the analyte, and this method can also be used for the pharmacokinetic study of voxtalisib, which can provide reference for the optimization of clinical drug management in the later period.

## Introduction

**FIGURE 1:**
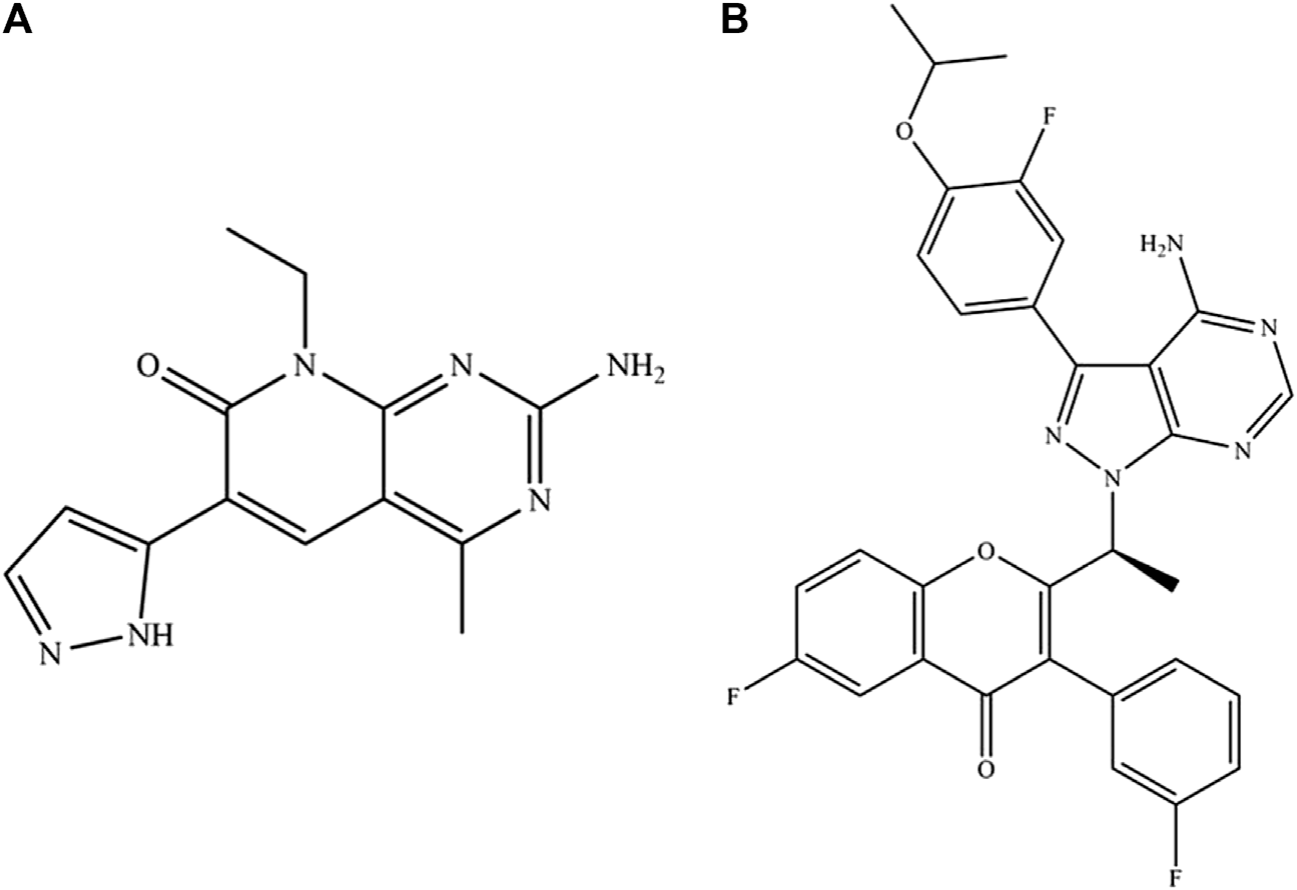
The structures of voxtalisib **(A)** and umbralisib **(B)**.

Voxtalisib (Figure 1A), also called as SAR245409 or XL765, is a dual inhibitor that inhibits both pan-class I phosphoinositide 3-kinase (PI3K) and mechanistic target of rapamycin (mTOR) by competitively binding specific adenosine triphosphate (ATP) to the catalytic domain of PI3K and mTOR (Janne et al., 2014; He et al., 2018). Voxtalisib plays an anti-tumor role mainly by inhibiting the formation of tumor blood vessels and inducing apoptosis of cancer cells, and its anti-tumor activity has been proved (Markman et al., 2010; Prasad et al., 2011; Zhao et al., 2019). Voxtalisib achieved its therapeutic effect by apoptotic caspase-dependent primary chronic lymphocytic leukemia cells with a half-maximum inhibitory concentration (IC_50_) of 0.86 µM and a maximum duration of action of 48 h. In addition, voxtalisib blocked the adhesion, proliferation, and *in vitro* survival of chronic lymphocytic leukemia cells and effectively inhibits T-cell-mediated cytokines that support the production of chronic lymphocytic leukemia (Thijssen et al., 2016; Brown et al., 2018; Zhang et al., 2018). As pharmacokinetic information is essential for the optimization of clinical administration, it is necessary to quantify and monitor the plasma concentration of voxtalisib.

Therefore, in this experiment, we intended to measure the concentration of voxtalisib in rat plasma by using a high-efficiency UPLC-MS/MS technology. Then, we demonstrated the robustness of our method by measuring the linearity, carryover, precision and accuracy, matrix effect and extraction recovery, and stability of the analyte. Subsequently, the pharmacokinetic studies of voxtalisib in rat plasma confirmed the specificity and repeatability of this method.

## Experiment

### Chemical Materials

Voxitalisib, and umbralisib (Figure 1B) as the internal standard (IS) were purchased from Beijing sunflower Technology Development Co., Ltd. (Beijing, China). The LC grade methanol and acetonitrile were supplied by Merck (Darmstadt, Germany) and LC grade pure formic acid was produced by Anaqua Chemicals Supply (ACS, American). At the same time, the ultra-pure water from the laboratory was pretreated by the Milli-Q Water Purification System (Millipore, Bedford, Unnited States).

### Animal Experiments

Six male SD rats (300 ± 20 g) were collected from the Experimental Animal Research Center of The First Affiliated Hospital of Wenzhou Medical University (Wenzhou, China), and were given fresh water and plenty of food every day. In the formal pharmacokinetic study, each rat was given 5 mg/kg voxtalisib in the 0.5% carboxymethylcellulose sodium (CMC-Na) by single intragastric administration after fasting for 12 h. Then, blood samples (approximately 0.3 ml) were acquired at 0.333, 0.667, 1, 1.5, 2, 3, 4, 6, 8, 12, 24 and 48 h, respectively, and placed into polyethylene tubes containing heparin. The samples were immediately centrifuged at 13,000 × g for 10 min at 4°C for separation. The centrifuged supernatants were then transferred into new 0.5 ml polythene tubes and then stored at −80°C for further analysis. In this experiment, the plasma concentration of voxtalisib in rats was measured by UPLC-MS/MS technology, and the main pharmacokinetic parameters of voxtalisib in non-compartmental model were calculated using Drugs and Statistics (DAS) 3.0 software (Mathematical Pharmacology Professional Committee of China, Shanghai, China).

### Analytical Conditions of the Instrumentation

Chromatographic separation of voxtalisib and IS were performed using a Waters Acquity ultra-performance liquid chromatography (UPLC) system (Milford, MA, Unnited States) equipped with an Acquity BEH C18 column (2.1 mm × 50 mm, 1.7 μm; Milford, MA, Unnited States), and it was operated under the condition of 40°C column temperature. The mobile phases of gradient elution were acetonitrile (solvent A) and 0.1% formic acid (solvent B), respectively. At the flow rate of 0.30 ml/min, the elution process was 90% B from 0 to 0.5 min; 90–10% B from 0.5 to 1.0 min; 10% B from 1.0 to 1.4 min; 10–90% B from 1.4 to 1.5 min, and finally at 1.5–2.0 min, 90% B was maintained for equilibration. The whole process of analysis was 2.0 min, the temperature of the auto-sampler was set at 10°C, and the injection volume was 1.0 μL for each running process.

**FIGURE 2:**
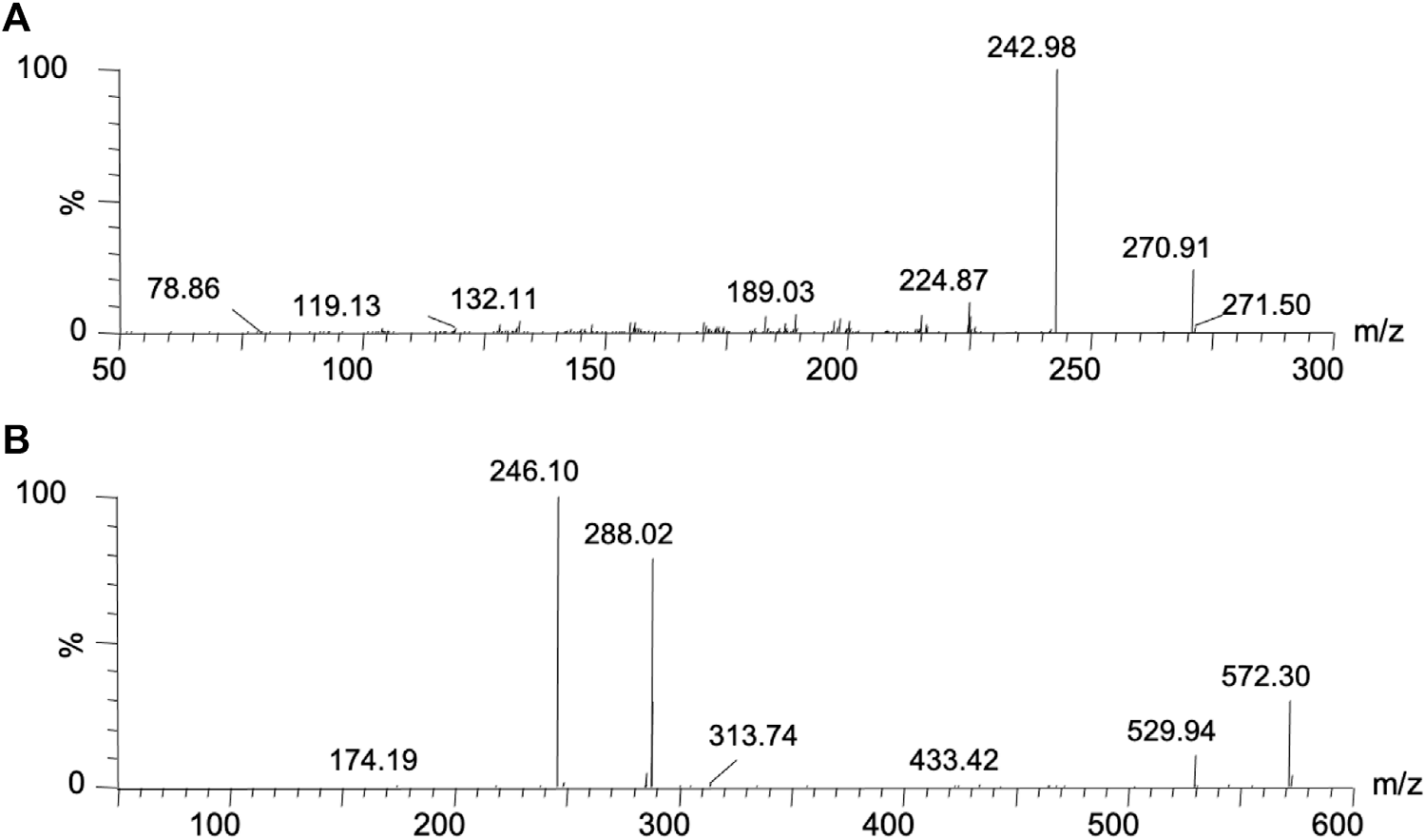
Mass spectrum of voxtalisib **(A)** and umbralisib **(B)** in this study.

Mass spectrometry information of voxtalisib and IS were obtained by a Waters Xevo TQS triple-quadrupole tandem mass spectrometer (Milford, MA, Unnited States) with an electrospray ionization (ESI) source and a positive ion mode detection by MS/MS. Using selective response monitoring (SRM), the ion transitions of voxtalisib and IS were *m/z* 270.91 > 242.98 and *m/z* 572.30 > 246.10, respectively (Figure 2). Optimized cone voltages and collision energies were 20 V and 20 eV for voxtalisib, 30 V and 35 eV for IS, respectively. The flow rate of collision gas, cone gas and desolvation gas filled with high purity of nitrogen was 0.15 ml/min, 200 L/h and 1000 L/h, respectively. The optimized desolvation temperature reached 600°C.

### Stock Solutions, Calibration and Quality Control Samples

Voxtalisib and IS were dissolved in methanol as the stock solutions, and the concentration of each stock solution was 1 mg/ml. Then, the stock solution was gradient diluted with methanol to obtain the calibration and quality control (QC) samples. The concentrations of the working solutions for calibration curve were 10, 20, 50, 100, 500, 1000, 2000, 5000, 10000, 20000 ng/ml, and the IS concentration was 100 ng/ml. Fresh calibration standards with final concentrations of 1, 2, 5, 10, 50, 100, 200, 500, 1000, 2000 ng/ml were prepared by adding the corresponding concentration of voxtalisib working solutions (10 μL) to the blank rat plasma (90 μL). Then, the same method was used to prepare QC samples with different concentrations (2, 800, 1600 ng/ml) and the lower limit of quantification (LLOQ, 1 ng/ml). Finally, all the stock and working solutions were stored at −80°C until further analysis.

### Sample Preparation

The plasma samples were treated with a simple and rapid protein precipitation method. Firstly, adding 10 μL IS working solution to 100 μL plasma sample; Secondly, precipitate the plasma protein by adding 300 μL acetonitrile; Thirdly, the plasma samples in each tube were fully swirled for 2.0 min and centrifuged for 10 min at 13000 × g under 4°C. Finally, we took 100 μL centrifuged supernatant into the special detection bottle of UPLC-MS/MS system, and then used UPLC-MS/MS technology for analysis. In order to ensure the stability of the sample, it is recommended to test immediately. If immediate detection is not available, it can be stored at 4°C in the short-term, and should be stored at −80°C in the long-term.

### Method Valiadation

According to the principle of bio-assay validation of FDA, a novel bio-assay method based on UPLC-MS/MS technology was established, including the specificity, carryover, calibration curve, LLOQ, precision, accuracy, matrix effect, recovery, and stability, as similar with those validated in our previous papers (Qiu et al., 2019; Xu et al., 2019; Tang et al., 2020).

### Specificity and Carryover Effect

The specificity of UPLC-MS/MS analytical method was determined by analyzing plasma samples from three different batches: blank plasma without analyte and IS, blank plasma spiked with the analyte at LLOQ and IS, as well as rat plasma samples. Then, the retention times of the analyte and IS in the SRM chromatogram were observed to determine whether there were endogenous interference. Carryover was assessed by injecting plasma samples at upper limit of quantification (ULOQ) followed by blank injections.

### Calibration Curve Linearity and LLOQ

The linear regression analysis of voxtalisib/IS peak area ratio (Y) and voxtalisib concentration (X) was performed by the least square regression analysis with a weighted factor (1/*x* ^2^) to obtain the calibration curve. The sensitivity of the UPLC-MS/MS bio-analytical method was evaluated by analyzing six replicates of spiked LLOQ samples.

### Precision and Accuracy

Six replicate QC samples (2, 800 and 1600 ng/ml) were determined on the same day and on three different days. The intra and inter-day precisions of the method were calculated, which is usually shown as relative standard deviation (RSD%), and the accuracy is commonly represented by relative error (RE%).

### Matrix Effect and Recovery

At low, medium and high concentrations (2, 800 and 1600 ng/ml, *n* = 6), the peak areas of voxtalisib added to the post-extracted blank plasma were compared with those of QC sample with corresponding concentration obtained by diluting standard solution with methanol to calculate matrix effect.

Voxtalisib was added into the blank plasma before and after extraction, respectively, and the extraction recovery was evaluated by comparing the peak area of the two, also at three different levels (2, 800 and 1600 ng/ml, *n* = 6).

### Stability

We evaluated the stability of this bioassay by measuring rat plasma concentrations of QC samples of voxtalisib at three different concentrations (2, 800 and 1600 ng/ml, *n* = 5) under four different placement conditions, including short-term stability (3 h at room temperature), long-term stability (3 weeks at −80°C), post-preparation stability (4 h at 10°C in an auto-sampler), and stability through complete three freeze-thaw cycles.

## Results

### The Optimal Condition of UPLC-MS/MS

In the pre-experiment, different organic buffers (such as methanol and acetonitrile) and water buffers (such as formic acid and ammonium acetate) on different types of analytical columns (Waters Acquity UPLC BEH C18 column, HSS C18 column, and CSH C18 column) were evaluated. After comparing different types of analytical columns in the experiment, the Acquity BEH C18 column (2.1 mm × 50 mm, 1.7 μm) provided good peak shapes, short chromatographic retention times and high chromatographic responses for both the analyte and IS, and the separation effect was better. In addition, acetonitrile and 0.1% formic acid aqueous solution was the best mobile phase, and the analyte and IS obtained better signals and responses.

In pharmacokinetic study, we chose simple, time-saving and economical organic protein precipitation method to prepare plasma samples. We precipitated plasma proteins with acetonitrile. The results showed that acetonitrile had higher efficiency of protein precipitation.

### Validation of the Method

#### Specificity and Carryover

**FIGURE 3:**
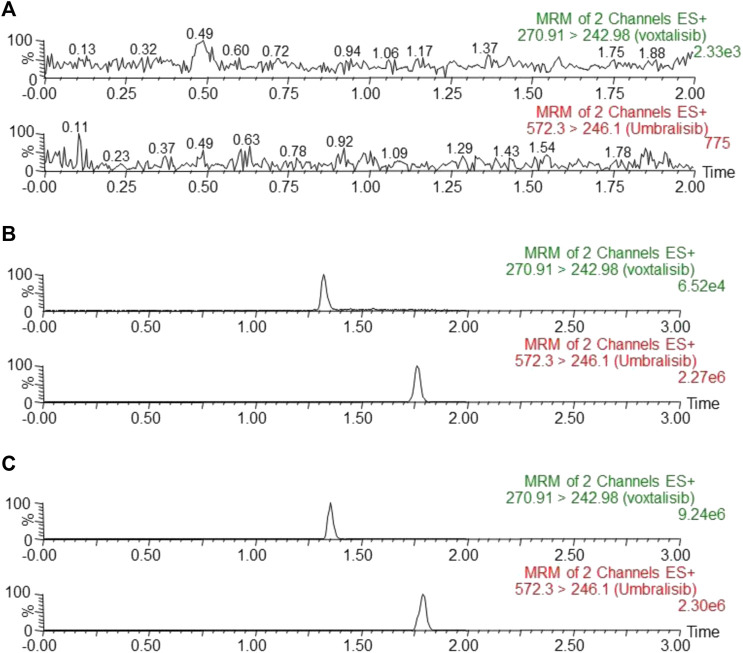
Representative chromatograms of voxtalisib and IS in rat plasma: **(A)** blank plasma; **(B)** blank plasma spiked with analyte at LLOQ and IS; **(C)** plasma sample collected from a rat at 20 min after intragastric administration of 5 mg/kg voxtalisib.

According to SRM chromatograms under three different conditions in Figure 3, voxtalisib and IS can be significantly distinguished by this chromatographic condition. The three different conditions were as follows: blank rat plasma sample (Figure 3A: no analyte, no IS), LLOQ concentration (1 ng/ml) of the analyte and IS added to blank rat plasma sample (Figure 3B), and a rat sample collected 20 min later in pharmacokinetic study after given intragastric administration of voxtalisib (5 mg/kg) (Figure 3C). Figure 3 showed that the retention times of voxtalisib and IS were 1.32 and 1.77 min, respectively. In addition, no carryover was observed for either analyte or IS in rat plasma, because the peak area of the interference peak was less than 20% for the analyte in the LLOQ samples and less than 5% for the IS following injection of ULOQ samples.

#### Linearity and LLOQ

In this experiment, the linear regression equation of calibration standard curve in the concentration range of 1–2000 ng/ml was y = 0.0231301x + 0.00967189 (*r* ^2^ = 0.999). As shown in Table 1, the LLOQ of voxtalisib was 1 ng/ml, where precision and accuracy were acceptable within 20%.

**TABLE 1:** The precision and accuracy of voxtalisib in rat plasma (*n* = 6).

Analyte	Concentration (ng/ml)	Intra-day		Inter-day	
		RSD (%)	RE (%)	RSD (%)	RE (%)
Voxtalisib	1	18.7	2.0	16.6	3.1
	2	10.6	−14.0	14.0	−4.5
	800	8.7	−4.0	14.3	−4.4
	1600	7.5	−8.5	13.0	−7.2

#### Precision and Accuracy

Table 1 indicated that the intra and inter-day precision values (RSD%) of voxtalisib at three QC concentrations were 7.5–10.6% and 13.0–14.3%, respectively, and the intra and inter-day accuracy values (RE%) were −14.0% to −4.0% and −7.2% to −4.4%, respectively. According to the FDA guidance, this bioassay was used to quantify voxtalisib in rat plasma with high accuracy and reproducibility.

#### Matrix Effect and Extraction Recovery

Table 2 displayed the results of matrix effect and extraction recovery of QC samples of voxtalisib at three different concentration levels (2, 800, 1600 ng/ml). The matrix effect of voxtalisib in blank rat plasma was 94.1–96.7%. In addition, its extraction recovery ranged from 100.5 to 106.8%. And these results were within the reasonable limits.

**TABLE 2:** Recovery and matrix effect of voxtalisib in rat plasma (*n* = 6).

Analyte	Concentration (ng/ml)	Recovery (%)		Matrix effect (%)	
		Mean ± SD	RSD (%)	Mean ± SD	RSD (%)
Voxtalisib	2	100.5 ± 8.6	8.5	94.1 ± 12.4	13.2
	800	106.8 ± 4.2	3.9	94.2 ± 3.7	3.9
	1600	105.1 ± 10.2	9.7	96.7 ± 5.7	5.9

#### Stability

Table 3 showed the stability results of QC samples of voxtalisib in rat plasma under four different placement conditions. These indicated that voxtalisib had no significant difference under a variety of placement conditions (3 h at room temperature, 3 weeks at −80°C, 4 h at 10°C in an auto-sampler, and freeze–thaw stability for 3 times).

**TABLE 3:** Stability results of voxtalisib in rat plasma under different conditions (*n* = 5).

Analyte	Concentration (ng/ml)	Room temperature, 3 h		Auto-sampler 10°C, 4 h		Three freeze-thaw		−80°C, 3 weeks	
		RSD (%)	RE (%)	RSD (%)	RE (%)	RSD (%)	RE (%)	RSD (%)	RE (%)
Voxtalisib	2	8.5	2.0	4.4	−1.8	8.2	2.2	8.2	7.4
	800	5.5	14.6	4.5	7.0	2.4	10.6	3.8	0.9
	1600	5.1	7.1	5.1	4.6	3.8	9.5	6.5	−10.8

#### Pharmacokinetic Study in Rats

**FIGURE 4:**
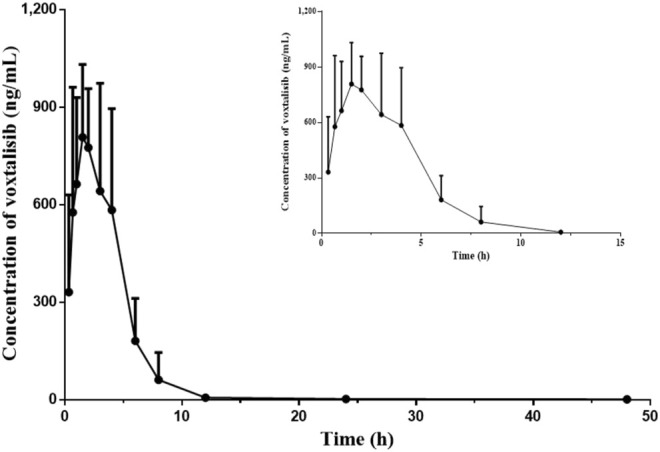
Mean plasma concentration-time curves of voxtalisib in rats after intragastric administration of voxtalisib (5 mg/kg). (*n* = 6).

The pharmacokinetic study of voxtalisib in rat plasma was performed by UPLC-MS/MS technology. Figure 4 depicted the relationship between mean plasma concentration (ng/ml) and time (0–48 h) of voxtalisib in rats. Since voxtalisib was fully eliminated from the body at around 12 h, the relationship between the mean plasma concentration (ng/ml) of voxtalisib in rats and the time of 0–12 h was added to illustrate the relationship more clearly. Table 4 showed the main pharmacokinetic parameters of voxtalisib, which were calculated by non-compartment analysis using DAS software.

**TABLE 4:** The main pharmacokinetic parameters of voxtalisib in rat plasma after intragastric administration of voxtalisib at a single dose of 5 mg/kg. (*n* = 6, Mean ± SD).

Parameters	Voxtalisib
AUC_0-t_ (ng/mL*h)	3762.79 ± 1165.22
AUC_0-∞ (_ng/mL*h)	3785.27 ± 1175.14
MRT_0-t_ (h)	3.77 ± 0.30
MRT_0-∞_ (h)	4.23 ± 0.20
t_1/2_ (h)	10.47 ± 5.00
T_max_ (h)	2.45 ± 1.42
CL_z_/F (L/h/kg)	1.47 ± 0.58
C_max_ (ng/ml)	977.74 ± 250.40

AUC_0-t_, area under the curve from 0 to t; AUC_0-∞_, area under the curve from 0 to infinity; MRT_0-t_, mean residence time from 0 to t; MRT_0-∞_, mean residence time from 0 to infinity; t_1/2_, elimination half-life; C_max_, peak plasma concentration; T_max_, time to C_max_; CL_z_/F, clearance.

According to Figure 4 and Table 4, after the rats were given 5 mg/kg voxtalisib in a single dose, voxtalisib was rapidly absorbed into the blood and spread throughout the whole body, reaching a maximum plasma concentration (C_max_) of 977.74 ± 250.40 ng/ml at 2.45 ± 1.42 h (time to C_max_, T_max_). Moreover, the half-life (t_1/2_) of its elimination in rats was 10.47 ± 5.00 h.

## Discussion

In a phase I dose-escalation study on the safety and pharmacokinetics of voxtalisib tablets in patients with solid tumors, C_max_ = 301 ± 101 ng/ml, T_max_ = 1.53 h, t_1/2_ = 3.94 ± 0.79 h on the first day after giving patients 50 mg/kg once daily (Mehnert et al., 2018). The existence of these differences in patient and rat data may be related to ethnic and individual differences, and in addition, our experimental results have only been verified in a few rats (*n* = 6), compared to 49 patients in the clinical trial. Consequently, the pharmacokinetics of voxtalisib need to be further studied. Furthermore, this pharmacokinetics of voxtalisib in patients with solid tumors did not provide enough data for repeating the approach in other laboratories (specificity, accuracy, precision, etc.), so our method effectively achieved the need of high sample throughput for biological analysis (Mehnert et al., 2018).

## Conclusion

In summary, in this experiment, we determined the specificity, carryover, precision, accuracy, extraction recovery, matrix effect, and stability of voxtalisib in rat plasma. This UPLC-MS/MS assay can effectively and quickly quantify the analyte, and this method can also be used for the pharmacokinetic study of voxtalisib in rats, which can provide reference for the optimization of clinical drug management in the later period.

## Data Availability

The original contributions presented in the study are included in the article/Supplementary Material, further inquiries can be directed to the corresponding authors.
